# Mean platelet volume and lymphocyte-to-monocyte ratio are associated with shorter progression-free survival in EGFR-mutant lung adenocarcinoma treated by EGFR tyrosine kinase inhibitor

**DOI:** 10.1371/journal.pone.0203625

**Published:** 2018-09-07

**Authors:** Kousuke Watanabe, Atsushi Yasumoto, Yosuke Amano, Hidenori Kage, Yasushi Goto, Yutaka Yatomi, Daiya Takai, Takahide Nagase

**Affiliations:** 1 Department of Respiratory Medicine, The University of Tokyo Hospital, Hongo, Bunkyo-ku, Tokyo, Japan; 2 Department of Clinical Laboratory, The University of Tokyo Hospital, Hongo, Bunkyo-ku, Tokyo, Japan; The Ohio State University, UNITED STATES

## Abstract

**Background:**

A growing body of evidence supports the role of platelets in cancer metastasis, escape from immune surveillance, and angiogenesis. Mean platelet volume (MPV), which reflects platelet turnover, is reported routinely as part of automated complete blood count. Accumulating evidence suggests that MPV is a useful biomarker in several diseases including cancer. However, its role in cancer patients receiving molecular targeted therapy has not been described in the literature.

**Materials and methods:**

We retrospectively analysed the prognostic impact of MPV in advanced or recurrent EGFR mutant lung adenocarcinoma treated with EGFR tyrosine kinase inhibitors (EGFR-TKIs). Lymphocyte-to-monocyte ratio (LMR) has been previously reported to be a poor prognostic factor in EGFR mutant non-small cell lung cancer and was also included as a covariate.

**Results:**

Using the previously described Cutoff Finder algorithm, the cut-off points for MPV and LMR that best predicted progression free survival (PFS) of EGFR-TKI were determined as 10.3 and 2.8, respectively. The median PFS was 14.7 and 8.2 months in MPV low and high groups (p = 0.013, log-rank test). The median PFS was 13.5 and 6.2 months in LMR high and low groups (p < 0.001, log-rank test). MPV and LMR were independently distributed (chi square test) and the multivariate analysis using Cox’s proportional hazards regression model revealed that high MPV, low LMR, and pleural effusion were significant predictors for shorter PFS.

**Conclusion:**

MPV and LMR, measured as part of routine complete blood count, can be utilized to predict the outcome of EGFR-TKI therapy with no additional costs. Our results suggest a mechanism of EGFR-TKI resistance which is associated with the functional status of the platelets.

## Introduction

Platelets have been increasingly recognized as having functional roles in the pathogenesis of cancer [1]. Platelets are activated by cancer cells through direct cellular contact or through the mediators released from cancer cells. The activated platelets in turn induces phenotypic changes of cancer cells such as metastasis and angiogenesis. Thus, functional status of platelets is a potential cancer biomarker.

Circulating platelets are heterogeneous in size and mean platelet volume (MPV) is the average volume of the circulating platelets. MPV is routinely reported as part of automated complete blood count. As younger platelets are larger and metabolically and enzymatically more active [2], increased MPV indicates increased platelet turnover and activity [3].

MPV is a simple indicator of the functional status of the platelets and high MPV is a prognostic factor in cardiovascular disease [4,5]. MPV is also a biomarker for inflammatory diseases such as ankylosing spondylitis, rheumatoid arthritis and inflammatory bowel disease [6,7].

The role of MPV in cancer is not fully understood. A recent systematic review of 18 studies showed that MPV is increased in patients with cancer other than lung cancer [8]. In lung cancer, however, the data are conflicting. Low MPV is associated with a shorter disease-free survival (DFS) and overall survival (OS) in patients with early stage non-small cell lung cancer (NSCLC) undergoing surgery [9]. It is also reported that MPV is decreased in advanced NSCLC and the decreased MPV/platelet count ratio is an unfavourable prognostic factor [10]. In contrast, another study shows that MPV is increased in advanced NSCLC and high MPV is associated with worse prognosis [11]. These studies analysed unselected NSCLC patients and the conflicting results may be due in part to the heterogeneity of NSCLC, with different somatic mutations [12].

No report has described the role of MPV in patients undergoing molecular targeted therapy in any type of cancer and EGFR mutation is the most common targetable oncogenic driver in NSCLC [12]. Interestingly, circulating platelets contain growth factors and cytokines that have been shown to induce resistance to EGFR tyrosine kinase inhibitor (EGFR-TKI). Platelets contain HGF [13] and TGF-β [14] and these molecules can induce resistance to EGFR-TKI through the activation of bypass signalling pathway [15] or the induction of epithelial to mesenchymal transition [16], respectively. Therefore, the present study focused on the role of MPV in EGFR mutation positive lung adenocarcinoma who received EGFR-TKI.

In addition to MPV, we examined lymphocyte-to-monocyte ratio (LMR) as another parameter of automated complete blood count. Decreased LMR is reported as a poor prognostic factor in several malignancies including EGFR-mutant NSCLC receiving EGFR-TKIs [17]. Within the tumor microenvironment, cytotoxic T lymphocytes play important roles in immune response against cancer and macrophages originating from the circulating monocytes are associated with tumor progression [18]. LMR is a potential biomarker of tumor microenvironment and host immunity. We investigated the impact of MPV and LMR on the prognosis of EGFR-mutant lung adenocarcinoma treated with EGFR-TKI.

## Materials and methods

### Patient cohort

We retrospectively reviewed all the advanced or recurrent NSCLC patients with common EGFR mutation (either L858R point mutation or exon 19 deletion) treated with EGFR-TKI between 2008 and 2017 at the University of Tokyo hospital. Patients with active infection, systemic corticosteroids, or haematological malignancy were excluded because these conditions might affect the values of haematological laboratory data. The patients with *de novo* T790M mutation (mechanism of TKI resistance), histology other than adenocarcinoma and early (within two months) discontinuation of TKI due to adverse events were also excluded. In this retrospective study, all data were fully anonymized before we accessed them. The study was approved by the institutional review board of the University of Tokyo hospital (approval number: 2739–5) and the need for informed consent was waived because of the retrospective and non-interventional design of the study.

### Data extraction

The following variables were extracted: haematological data (MPV, LMR, lymphocyte count, monocyte count, and platelet count), Eastern Cooperative Oncology Group performance status (ECOG PS), age, EGFR mutation (L858R or exon 19 deletion), Glasgow Prognostic Score (GPS), smoking, treatment line, metastatic lesions at the time of initiation of TKI, prior therapy such as surgery or radiotherapy, and the choice of TKI (gefitinib, erlotinib, or afatinib). GPS is an inflammation-based cancer prognostic factor, and was calculated from the values of C-reactive protein (CRP) and albumin as described in the literature [19]. Patients with both hypoalbuminemia and increased CRP were allocated a score of 2, patients with either hypoalbuminemia or increased CRP were allocated a score of 1, and patients with neither were allocated a score of 0. All laboratory data were obtained at a single laboratory within one month (usually within one week) before starting TKI therapy. Blood samples were taken in the presence of EDTA-K2 (ethylene diamine tetraacetic acid) and automated complete blood count was performed using the Sysmex XE or XN analyser (Sysmex, Kobe, Japan). LMR was calculated by dividing the lymphocyte count by the monocyte count.

### Statistical analysis

We applied the previously described Cutoff Finder algorithm [20] to determine the optimal cut-off points of MPV and LMR that best predicted progression free survival (PFS) of EGFR-TKI. Cutoff finder is a web-based application programmed in R (http://molpath.charite.de/cutoff). In this application, continuous variables such as MPV and LMR were dichotomized at each possible cut-off points and Cox’s proportional hazards regression models were applied to the dichotomized variable and survival data. Survival analysis is performed using the R package “survival” and the optimal cut-off point is defined as the point with the most significant (log-rank test) split.

The primary endpoint was PFS calculated from the date of the TKI start to documented disease progression or death from any cause. The secondary endpoint was overall survival (OS) calculated from the date of the TKI start to death from any cause. PFS and OS were estimated using the Kaplan-Meier method and were compared by the log-rank test. Prognostic factors for PFS and OS were assessed by the Cox’s proportional hazards regression model.

The multivariate Cox’s proportional hazards regression model was validated using two-step bootstrap procedures [21]. In the first step, 300 bootstrap samples were generated randomly with replacement from the study population to validate variable selection. A stepwise procedure was applied to each sample using a significance level of 0.15 for entering and removing explanatory variables. The factors that were selected in more than 50% of the models were considered significant. In the second step, 300 bootstrap samples were created to validate the hazard ratio (HR) estimate of the Cox’ proportional hazards regression model. For each bootstrap sample, we performed multivariate Cox’s proportional hazard regression analysis using variables selected in the first step. The mean and the percentile 95% confidence interval (CI) of HR were computed from the 300 samples.

Finally, to analyse the impact of clinical parameters on MPV and LMR, comparisons between groups were performed using a Mann-Whitney U test or a Kruskal Wallis test for nonparametric variables and a Student’s t test or an analysis of variance (ANOVA) for parametric variables. Statistical analyses were performed using the packages “stats”, “survival”, “My.stepwise” and “exactRankTests” in the R software (version 3.5.1). All tests were two-tailed and p values lower than 0.05 were regarded as statistically significant.

## Results

### Patient characteristics

In the study period, 82 advanced or recurrent NSCLC patients with common EGFR mutation were treated with EGFR-TKI. Patients with active infection (n = 2), systemic corticosteroids (n = 2), haematological malignancy (n = 1), *de novo* T790M mutation (n = 1), histology other than adenocarcinoma (pleomorphic carcinoma, n = 1), and early discontinuation of TKI due to adverse events (n = 3) were excluded from the analysis, and a total of 72 patients were included in the analysis. Of the 72 cases, 52 had advanced disease not suitable for surgery or radiotherapy and the remaining 20 were recurrent cases after surgery or stereotactic radiotherapy (SRT). Most (n = 67) of the patients received TKI as first line therapy. The mean time (± standard deviation) from the acquisition of the laboratory data to TKI start was 2.7 ± 4.5 days. The maximum and minimum values of MPV were 8.4 and 12.4 fl respectively and most (n = 68) of the patients had MPV values within the institutional reference range (6.5–11.7 fl). The detailed clinical characteristics of the 72 patients are shown in Table 1.

**Table 1:** Patient characteristics.

Variables			N	%
Age years	Median		69	
	Range		35–86	
	<75		50	69
	≥75		22	31
Gender	Female		41	57
	Male		31	43
ECOG PS *	0		27	38
	1		31	43
	2		8	11
	3		5	7
	not available		1	1
Smoking	Never		38	53
	Ever		34	47
EGFR mutation	L858R		36	50
	Exon19 deletion		36	50
Treatment line	First line TKI**		67	93
	TKI after chemotherapy		5	7
Metastasis ***	Lymph node metastasis		38	53
	Pleural effusion		28	39
	Bone metastasis		27	38
	Brain metastasis		16	22
Stage ****	IIIA, not suitable for radiation or surgery		2	3
	IIIB		2	3
	IV		48	66
	Recurrence after surgery		18	25
	Recurrence after SRT*****		2	3

* Eastern Cooperative Oncology Group performance status

** Tyrosine kinase inhibitor

*** site(s) of metastatic spread at the time of initiation of TKI

**** 7th edition of TNM staging of lung cancer

***** Stereotactic radiotherapy

Using the previously described Cutoff Finder algorithm [20], the optimal cut-off points for MPV and LMR that best predicted the PFS were calculated to be 10.25 and 2.784, respectively (Parts A and B of S1 Fig). As these values were equivalent to defining MPV ≥ 10.3 and LMR ≤ 2.8 as risk factors for shorter PFS in our dataset, we determined the cut-off points for MPV and LMR as 10.3 and 2.8, respectively. The monocyte percentage was zero in one case and this case was classified as having high LMR. The distribution of the number of patients based on these cut-off values (Table 2) indicates that MPV and LMR are independent biomarkers (p = 0.564, chi square test). The Cutoff Finder algorithm was also applied to determine whether there is an optimal cut-off point for platelet count that predicts PFS, but none of the possible cut-off values achieved statistical significance (Part C of S1 Fig).

**Table 2:** Distribution of the number of patients.

	LMR		
	low(≤2.8)	high(>2.8)	sum, N (%)
**MPV**			
high(≥10.3), N	12	22	34 (47)
low (<10.3), N	11	27	38 (53)
sum, N (%)	23 (32)	49 (68)	72 (100)

p = 0.564 (chi square test)

### Survival analysis

**Fig 1:**
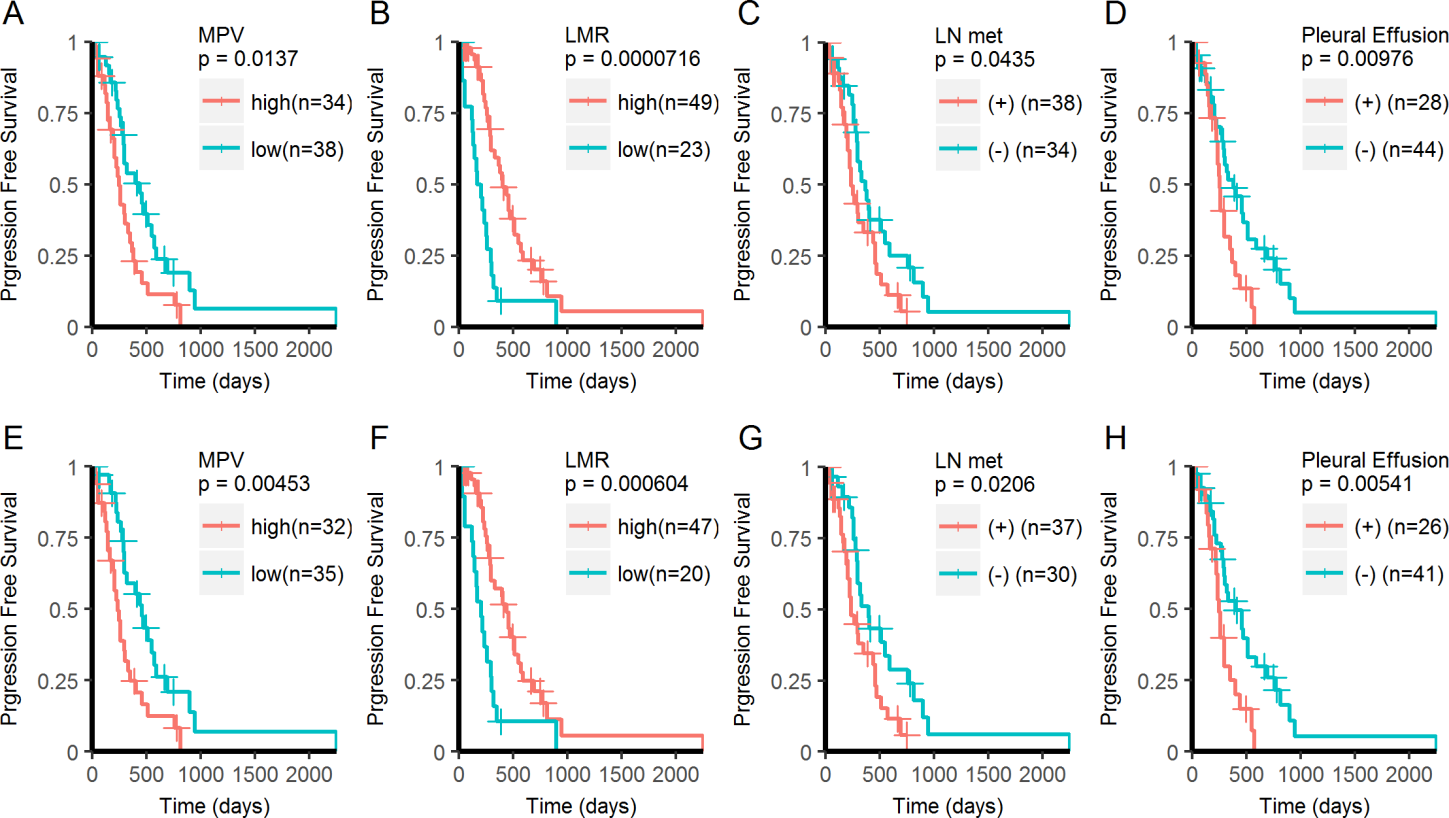
Kaplan-Meier analysis of PFS. (A-D) Kaplan-Meier curves for PFS in EGFR mutant lung adenocarcinoma treated with EGFR-TKI. (E-H) Kaplan-Meier curves for PFS in EGFR mutant lung adenocarcinoma receiving EGFR-TKI as first line therapy.

The median length of follow-up (Kaplan-Meier estimate) for PFS was 25.8 months (95% CI, 22.1-NA months). A total of 56 events out of 72 patients (77% of the patients) were observed during the follow-up period and the median PFS in all patients was 9.9 months (95% CI, 8.6–13.5 months). Univariate analysis using the Cox’s proportional hazards regression model showed that high MPV, low LMR, lymph node metastasis, and pleural effusion were significantly associated with shorter PFS (S1 Table). In the multivariate analysis using these four variables, high MPV, low LMR, and pleural effusion were significant predictors for shorter PFS (S1 Table). The prognostic impact of high MPV, low LMR, lymph node metastasis, and pleural effusion were also confirmed using log-rank test (Fig 1A–1D). The median PFS was 14.7 (95% CI, 9.7–19.6) and 8.2 (95% CI, 6.8–12.3) months in MPV low and high groups, respectively. The median PFS was 13.5 (95% CI, 9.9–18.3) and 6.2 (95% CI, 4.5–9.8) months in LMR high and low groups, respectively. These four factors were also prognostic in the subset of patients (n = 67) receiving TKI as first line therapy (Fig 1E–1H).

**Fig 2:**
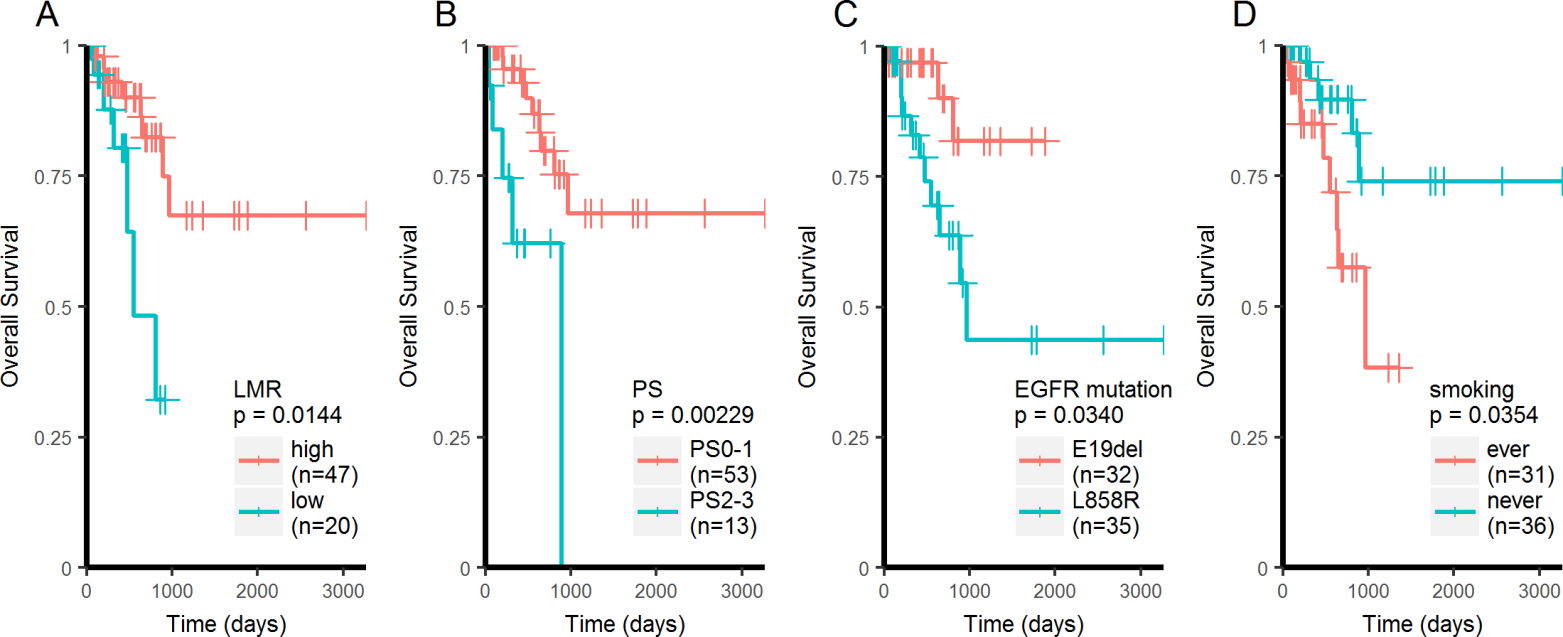
Kaplan-Meier analysis of OS. Kaplan-Meier curves for OS in in EGFR mutant lung adenocarcinoma receiving EGFR-TKI as first line therapy.

OS was calculated in the subset of patients receiving TKI as first line therapy (n = 67), but the median OS was not reached due to high censoring rate. Univariate analysis using Cox’s proportional hazards regression model showed that low LMR, poor PS, L858R mutation, and smoking history were significantly associated with shorter OS (S2 Table). Multivariate analysis using these four factors showed poor PS and smoking history as significant predictors for shorter OS (S2 Table). The prognostic impact of low LMR, poor PS, L858R mutation, and smoking history were also confirmed using log-rank test (Fig 2).

### Bootstrap validation

As the number of patients was small (n = 72), the prognostic impact of MPV and LMR on PFS was validated using two-step bootstrap procedures. In the first step, the stepwise Cox’s proportional hazards regression model was employed with each of the 300 random bootstrap samples of the same size (n = 72) drawn with replacement. The frequency of each variables to be selected in the stepwise model was calculated using 300 bootstrap samples (Part A of S3 Table). GPS was not included as a covariate because GPS data were not available in eight cases. As shown in Part A of S3 Table, four variables (LMR, MPV, poor PS and pleural effusion) were selected in more than 50% of the models. In the multivariate analysis using these four variables, all of them were significant predictors for shorter PFS (Part B of S3 Table).

In the second step, 300 bootstrap samples of the same size (n = 72) drawn with replacement were generated to validate the HR estimate of the regression model. For each bootstrap sample, we applied the Cox’s proportional hazards regression model using these four variables. The mean and the percentile 95% confidence interval (CI) of HRs calculated from the 300 samples (Part C of S3 Table) indicated that MPV and LMR were significant predictors for shorter PFS.

### Correlation of MPV and LMR with clinical parameters

**Fig 3:**
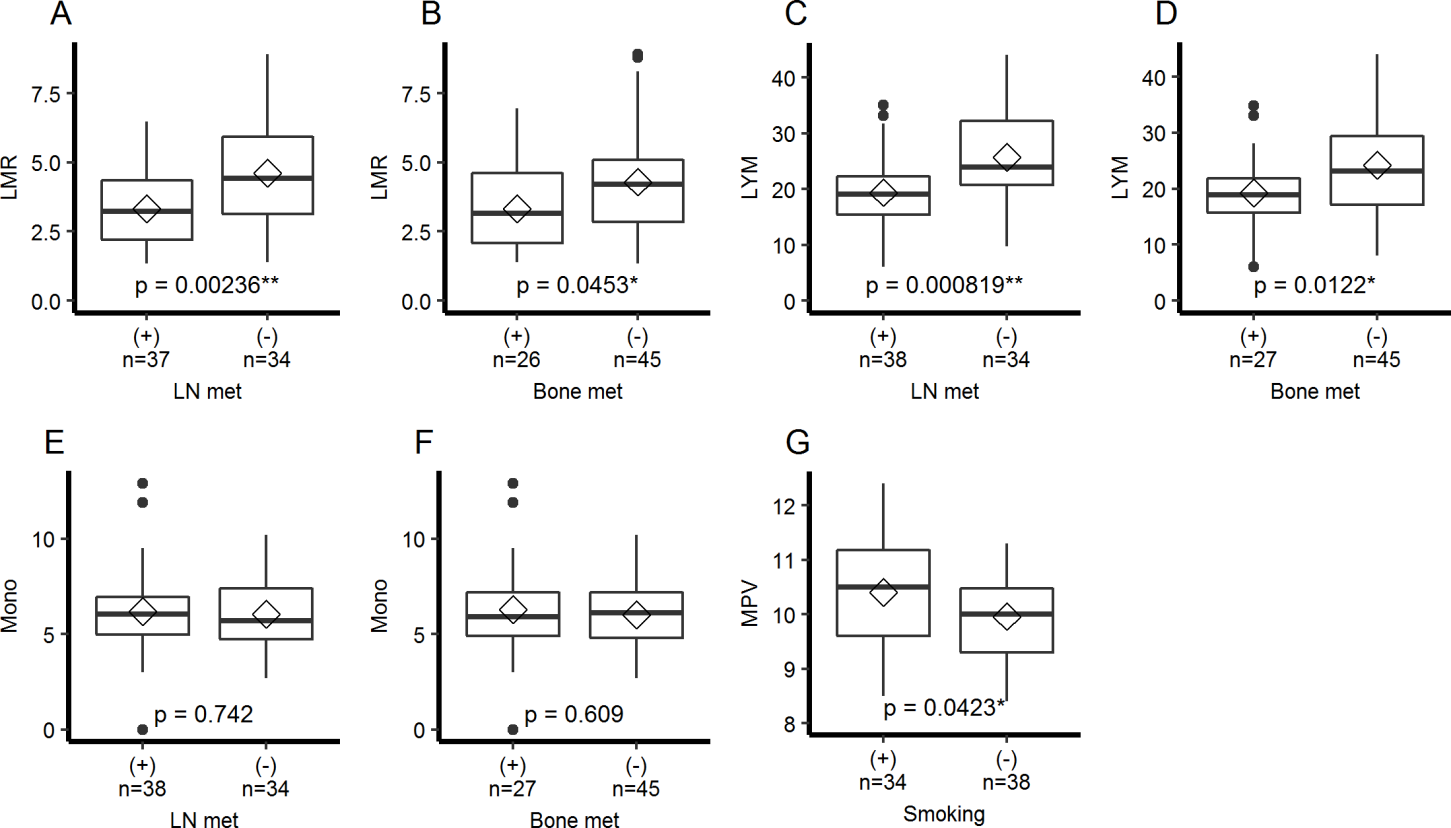
Association of LMR and MPV with metastasis and smoking. (A-F) LMR, lymphocyte percentage (LYM), and monocyte percentage (Mono) according to lymph node and bone metastasis. (G) MPV according to smoking history. As the monocyte percentage was zero in one case, this case was excluded from the analysis of LMR. Each boxplot indicates interquartile range (box), median (horizontal line), mean (diamond), minimum and maximum values within 1.5 times the interquartile range (whiskers), and outliers (points). Statistical comparisons between groups were performed using a Mann-Whitney U test for nonparametric variables (B, E) and a Student’s t test for parametric variables (A, C, D, F, and G). * p<0.05, ** p<0.01.

We analysed how the values of MPV and LMR differ according to clinical parameters (S4 and S5 Tables). LMR was significantly decreased in patients with lymph node metastasis or bone metastasis (Fig 3A and 3B). This difference reflected decreased lymphocyte percentage in these patients (Fig 3C and 3D) and no difference in monocyte percentage was detected (Fig 3E and 3F). MPV was not associated with metastasis but was significantly increased in patients with smoking history (Fig 3G).

**Fig 4:**
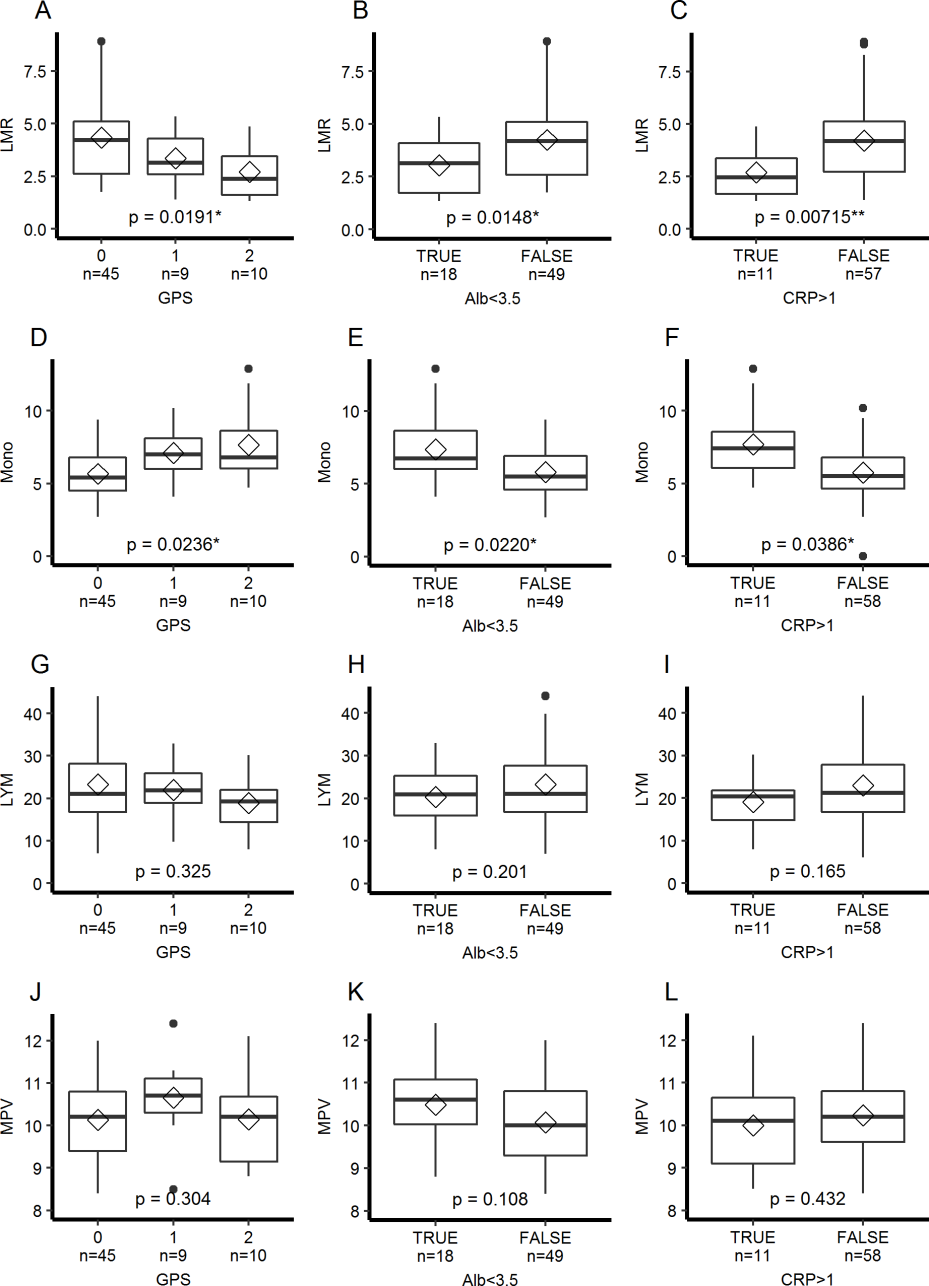
Association of LMR with Glasgow Prognostic score (GPS). LMR, lymphocyte percentage (LYM), and monocyte percentage (Mono) according to GPS. Boxplots are shown as in Fig 3. Statistical comparisons between groups were performed using a Mann-Whitney U test or a Kruskal Wallis test for nonparametric variables (A-D) and a Student’s t test or an analysis of variance (ANOVA) for parametric variables (E-L). * p<0.05, ** p<0.01.

We also investigated the impact of GPS, an inflammation-based cancer prognostic factor calculated from the values of CRP and albumin, on MPV and LMR (Fig 4). There was a significant relation between LMR and GPS (Fig 4A), and LMR was significantly decreased in patients with hypoalbuminemia or increased CRP (Fig 4B and 4C). This relationship was due to increased monocyte percentage in patients with hypoalbuminemia or increased CRP (Fig 4D–4F), and no difference was detected in lymphocyte percentage (Fig 4G–4I). In contrast, MPV was not associated with GPS, hypoalbuminemia or increased CRP (Fig 4J–4L).

**Fig 5:**
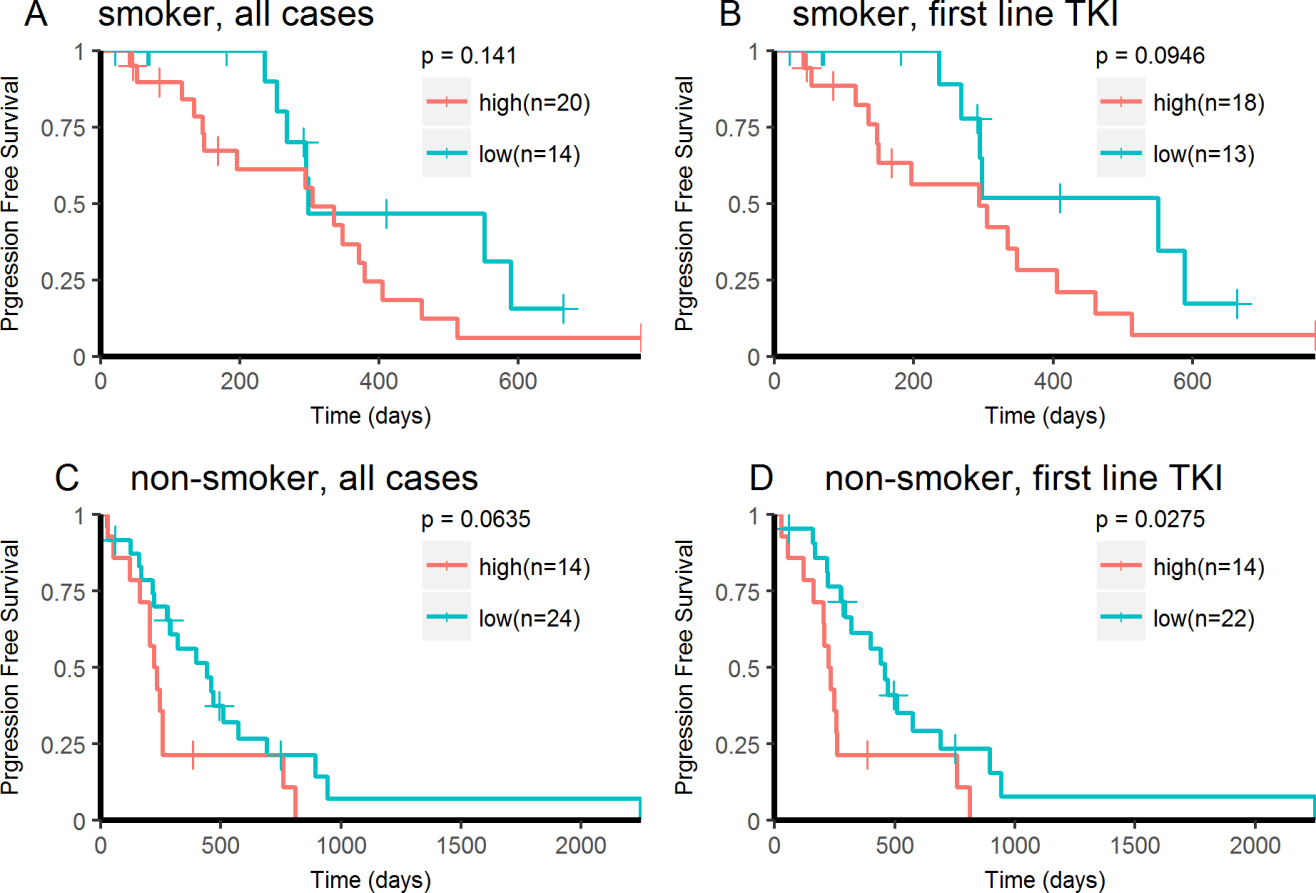
Kaplan-Meier analysis of PFS stratified by smoking history. Kaplan-Meier curves for PFS in EGFR mutant lung adenocarcinoma treated with EGFR-TKI in ever-smoker patients (A, B), and never smoker patients (C, D).

Having confirmed that MPV was significantly increased in patients with smoking history, we performed a subgroup analysis of PFS stratified by smoking history. Although statistically not significant, patients with high MPV tended to have shorter PFS in both ever-smokers and never smokers (Fig 5A and 5C). In never smokers receiving TKI as first line therapy, high MPV was significantly associated with shorter PFS (Fig 5D).

### MPV decreases during TKI therapy

**Fig 6:**
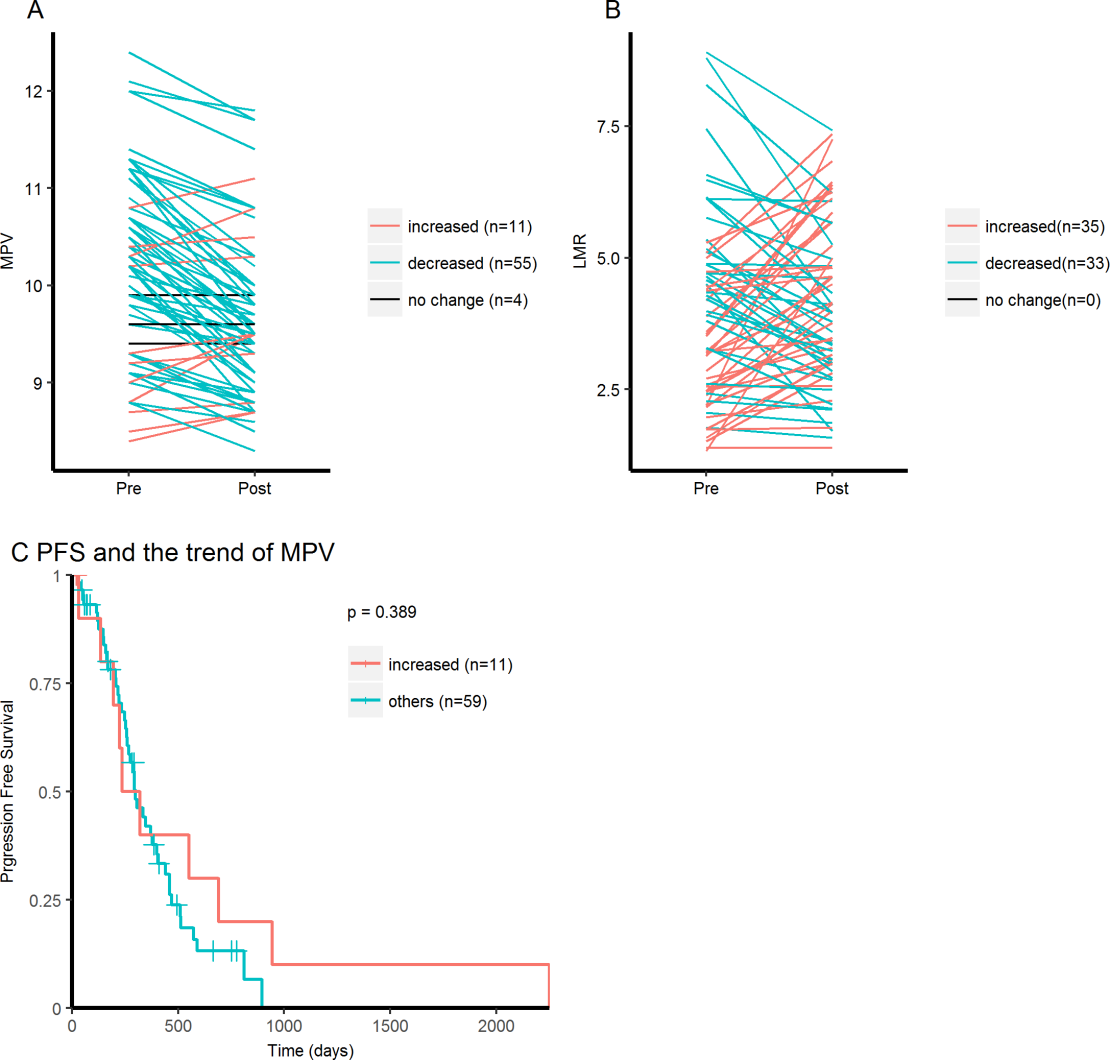
MPV and LMR before and after EGFR-TKI. (A, B) Comparison of MPV and LMR before TKI therapy with those after one month of TKI therapy. (C) Kaplan-Meier curves for PFS according to the trend of MPV.

The changes of MPV and LMR values in response to TKI therapy were analysed. We compared the values of MPV and LMR before TKI therapy with those after one month of TKI therapy. The comparison of MPV and LMR were available in 70 and 68 cases, respectively. MPV was significantly decreased after TKI therapy (Fig 6A) and the mean difference between pre-treatment and post-treatment was 0.51 (95% CI 0.38–0.63, p < 0.001, paired t test). In contrast, no significant difference between pre-treatment and post-treatment was detected in LMR (Fig 6B, p = 0.33, paired t test). We compared the PFS of patients whose MPV increased after one month of TKI therapy with the remaining patients, and no difference was detected (Fig 6C).

## Discussion

The present study demonstrated that high MPV, low LMR and pleural effusion were independent predictors for shorter PFS in EGFR-mutant lung adenocarcinoma treated with EGFR-TKI. Although paraneoplastic thrombocytosis is reported to be poor prognostic factor in many types of cancer including lung cancer [22,23], platelet count was not prognostic in EGFR-mutant lung adenocarcinoma treated with EGFR-TKI. MPV and LMR are measured as part of routine laboratory test and they can be utilized with no additional costs to predict the outcome of EGFR-TKI therapy. The prognostic role of LMR in EGFR-mutant NSCLC has been described in the literature [17]. To the best of our knowledge, this is the first report describing the prognostic role of MPV in EGFR-mutant NSCLC treated with EGFR-TKI.

We also showed that low LMR was associated with metastasis (lymph node and bone metastasis), malnutrition (hypoalbuminemia) and increased CRP. Therefore, the prognostic impact of LMR may be due to the confounding effect of metastasis, malnutrition and inflammation. In contrast, MPV was not associated with metastasis, malnutrition, or increased CRP, but was significantly increased in patients with smoking history. Since a meta-analysis has demonstrated that smoking is associated with shorter PFS and OS in patients receiving EGFR-TKI [24], we performed a subgroup analysis of PFS stratified by smoking history. Patients with high MPV tended to have shorter PFS in both smokers and non-smokers, and high MPV was significantly associated with shorter PFS in never smokers receiving TKI as first line therapy, suggesting that the prognostic impact of MPV is not the result of confounding effect of smoking.

The values of MPV was significantly decreased after one month of TKI therapy. It is not clear whether EGFR-TKI has a previously undescribed direct effect on platelets or the activation signal from cancer cells to platelets is decreased as a result of reduced tumor burden after EGFR-TKI. Further study is required to evaluate the change of MPV during treatment and its clinical implications.

There is a growing body of evidence that supports the role of platelets in cancer metastasis, escape from immune surveillance, and angiogenesis [1]. However, little is known whether platelets regulate the response to anti-cancer drugs, including cytotoxic chemotherapy, molecular targeted therapy, and immunotherapy. As for cytotoxic chemotherapy, the coculture of ovarian or colon cancer cells with platelets induced resistance to 5-fluorouracil and taxanes [25,26]. High MPV is associated with poor response to cytotoxic chemotherapy in advanced gastric cancer [27], suggesting that platelets induce resistance to cytotoxic chemotherapy. A recent study also demonstrated that platelets subvert T cell immunity against cancer in mouse [28] and it would be interesting to study whether platelet marker serves as a predictive marker in cancer immunotherapy. As platelets contain growth factors and cytokines such as HGF [13] and TGF-β [14] that have been shown to induce resistance to EGFR-TKI [15,16], further studies are required to clarify the role of platelets in EGFR-TKI resistance.

Our study has limitations. First, the cut-off point of MPV in the current study can be applied only when using Sysmex analysers, because haematological analysers from different constructors (Beckman Coulter, Siemens, and Sysmex) reportedly yield different MPV values [29]. Second, the high censoring rate at the end of the follow-up hampered accurate estimation of OS. Third, the study was retrospective with small number of patients and a prospective study including more patients is required to validate the results.

## Conclusions

MPV and LMR were independent predictors for shorter PFS in EGFR-mutant lung adenocarcinoma treated with EGFR-TKI. Our results suggest a novel mechanism of EGFR-TKI resistance which is associated with the functional status of platelets. Future studies are warranted to unravel the causal link between EGFR-TKI sensitivity and platelets.

## Supporting information

S1 Fig Determination of the optimal cut-off points using Cutoff Finder algorithm. Hazard ratio (HR) for PFS with 95% CI is plotted in dependence of MPV (A), LMR (B) and platelet count (C). The distribution of each case is shown as rug plot at the bottom of the figure. A vertical line designates the cut-off point showing the most significant correlation with PFS.
(TIF)

S1 Table Cox’s proportional hazards regression model for PFS. (XLSX)

S2 Table Cox’s proportional hazards regression model for OS. (XLSX)

S3 Table Bootstrap validation of multivariate Cox’s proportional hazards regression model for PFS. (XLSX)

S4 Table MPV values according to clinical parameters. (XLSX)

S5 Table LMR values according to clinical parameters. (XLSX)

## References

[1] Bambace NM, Holmes CE (2011) The platelet contribution to cancer progression. J Thromb Haemost 9: 237–249. 10.1111/j.1538-7836.2010.04131.x 21040448

[2] Karpatkin S (1969) Heterogeneity of human platelets. II. Functional evidence suggestive of young and old platelets. J Clin Invest 48: 1083–1087. 10.1172/JCI106064 5771189 PMC322322

[3] Gasparyan AY, Ayvazyan L, Mikhailidis DP, Kitas GD (2011) Mean platelet volume: a link between thrombosis and inflammation? Curr Pharm Des 17: 47–58. 21247392 10.2174/138161211795049804

[4] Chu SG, Becker RC, Berger PB, Bhatt DL, Eikelboom JW, Konkle B, et al (2010) Mean platelet volume as a predictor of cardiovascular risk: a systematic review and meta-analysis. J Thromb Haemost 8: 148–156. 10.1111/j.1538-7836.2009.03584.x 19691485 PMC3755496

[5] Slavka G, Perkmann T, Haslacher H, Greisenegger S, Marsik C, Wagner OF, et al (2011) Mean platelet volume may represent a predictive parameter for overall vascular mortality and ischemic heart disease. Arterioscler Thromb Vasc Biol 31: 1215–1218. 10.1161/ATVBAHA.110.221788 21330610

[6] Kisacik B, Tufan A, Kalyoncu U, Karadag O, Akdogan A, Ozturk MA, et al (2008) Mean platelet volume (MPV) as an inflammatory marker in ankylosing spondylitis and rheumatoid arthritis. Joint Bone Spine 75: 291–294. 10.1016/j.jbspin.2007.06.016 18403245

[7] Yuksel O, Helvaci K, Basar O, Koklu S, Caner S, Helvaci N, et al (2009) An overlooked indicator of disease activity in ulcerative colitis: mean platelet volume. Platelets 20: 277–281. 10.1080/09537100902856781 19459134

[8] Pyo JS, Sohn JH, Kang G (2016) Diagnostic and prognostic roles of the mean platelet volume in malignant tumors: a systematic review and meta-analysis. Platelets 27: 722–728. 10.3109/09537104.2016.1169265 27162007

[9] Kumagai S, Tokuno J, Ueda Y, Marumo S, Shoji T, Nishimura T, et al (2015) Prognostic significance of preoperative mean platelet volume in resected non-small-cell lung cancer. Mol Clin Oncol 3: 197–201. 10.3892/mco.2014.436 25469294 PMC4251106

[10] Inagaki N, Kibata K, Tamaki T, Shimizu T, Nomura S (2014) Prognostic impact of the mean platelet volume/platelet count ratio in terms of survival in advanced non-small cell lung cancer. Lung Cancer 83: 97–101. 10.1016/j.lungcan.2013.08.020 24189108

[11] Omar M, Tanriverdi O, Cokmert S, Oktay E, Yersal O, Pilanci KN, et al (2016) Role of increased mean platelet volume (MPV) and decreased MPV/platelet count ratio as poor prognostic factors in lung cancer. Clin Respir J. 10.1111/crj.12605 28026133

[12] Cancer Genome Atlas Research N (2014) Comprehensive molecular profiling of lung adenocarcinoma. Nature 511: 543–550. 10.1038/nature13385 25079552 PMC4231481

[13] Nakamura T, Teramoto H, Ichihara A (1986) Purification and characterization of a growth factor from rat platelets for mature parenchymal hepatocytes in primary cultures. Proc Natl Acad Sci U S A 83: 6489–6493. 3529086 10.1073/pnas.83.17.6489 PMC386529

[14] Assoian RK, Sporn MB (1986) Type beta transforming growth factor in human platelets: release during platelet degranulation and action on vascular smooth muscle cells. J Cell Biol 102: 1217–1223. 3457014 10.1083/jcb.102.4.1217 PMC2114151

[15] Yano S, Wang W, Li Q, Matsumoto K, Sakurama H, Nakamura T, et al (2008) Hepatocyte growth factor induces gefitinib resistance of lung adenocarcinoma with epidermal growth factor receptor-activating mutations. Cancer Res 68: 9479–9487. 10.1158/0008-5472.CAN-08-1643 19010923

[16] Suda K, Tomizawa K, Fujii M, Murakami H, Osada H, Maehara Y, et al (2011) Epithelial to mesenchymal transition in an epidermal growth factor receptor-mutant lung cancer cell line with acquired resistance to erlotinib. J Thorac Oncol 6: 1152–1161. 10.1097/JTO.0b013e318216ee52 21597390

[17] Chen YM, Lai CH, Chang HC, Chao TY, Tseng CC, Fang WF, et al (2015) Baseline and Trend of Lymphocyte-to-Monocyte Ratio as Prognostic Factors in Epidermal Growth Factor Receptor Mutant Non-Small Cell Lung Cancer Patients Treated with First-Line Epidermal Growth Factor Receptor Tyrosine Kinase Inhibitors. PLoS One 10: e0136252 10.1371/journal.pone.0136252 26313661 PMC4552380

[18] Speiser DE, Ho PC, Verdeil G (2016) Regulatory circuits of T cell function in cancer. Nat Rev Immunol 16: 599–611. 10.1038/nri.2016.80 27526640

[19] McMillan DC (2013) The systemic inflammation-based Glasgow Prognostic Score: a decade of experience in patients with cancer. Cancer Treat Rev 39: 534–540. 10.1016/j.ctrv.2012.08.003 22995477

[20] Budczies J, Klauschen F, Sinn BV, Gyorffy B, Schmitt WD, Darb-Esfahani S, et al (2012) Cutoff Finder: a comprehensive and straightforward Web application enabling rapid biomarker cutoff optimization. PLoS One 7: e51862 10.1371/journal.pone.0051862 23251644 PMC3522617

[21] Chen CH, George SL (1985) The bootstrap and identification of prognostic factors via Cox's proportional hazards regression model. Stat Med 4: 39–46. 3857702 10.1002/sim.4780040107

[22] Aoe K, Hiraki A, Ueoka H, Kiura K, Tabata M, Tanaka M, et al (2004) Thrombocytosis as a useful prognostic indicator in patients with lung cancer. Respiration 71: 170–173. 10.1159/000076679 15031573

[23] Lin RJ, Afshar-Kharghan V, Schafer AI (2014) Paraneoplastic thrombocytosis: the secrets of tumor self-promotion. Blood 124: 184–187. 10.1182/blood-2014-03-562538 24868077 PMC4093679

[24] Zhang Y, Kang S, Fang W, Hong S, Liang W, Yan Y, et al (2015) Impact of smoking status on EGFR-TKI efficacy for advanced non-small-cell lung cancer in EGFR mutants: a meta-analysis. Clin Lung Cancer 16: 144–151 e141. 10.1016/j.cllc.2014.09.008 25454007

[25] Bottsford-Miller J, Choi HJ, Dalton HJ, Stone RL, Cho MS, Haemmerle M, et al (2015) Differential platelet levels affect response to taxane-based therapy in ovarian cancer. Clin Cancer Res 21: 602–610. 10.1158/1078-0432.CCR-14-0870 25473001 PMC4315757

[26] Radziwon-Balicka A, Medina C, O'Driscoll L, Treumann A, Bazou D, Inkielewicz-Stepniak I, et al (2012) Platelets increase survival of adenocarcinoma cells challenged with anticancer drugs: mechanisms and implications for chemoresistance. Br J Pharmacol 167: 787–804. 10.1111/j.1476-5381.2012.01991.x 22506717 PMC3575779

[27] Lian L, Xia YY, Zhou C, Shen XM, Li XL, Han SG, et al (2015) Mean platelet volume predicts chemotherapy response and prognosis in patients with unresectable gastric cancer. Oncol Lett 10: 3419–3424. 10.3892/ol.2015.3784 26788144 PMC4665329

[28] Rachidi S, Metelli A, Riesenberg B, Wu BX, Nelson MH, Wallace C, et al (2017) Platelets subvert T cell immunity against cancer via GARP-TGFbeta axis. Sci Immunol 2. 10.1126/sciimmunol.aai7911 PMC5539882 28763790

[29] Latger-Cannard V, Hoarau M, Salignac S, Baumgart D, Nurden P, Lecompte T (2012) Mean platelet volume: comparison of three analysers towards standardization of platelet morphological phenotype. Int J Lab Hematol 34: 300–310. 10.1111/j.1751-553X.2011.01396.x 22225539

