# Supplementary figures and images for: Mean platelet volume and lymphocyte-to-monocyte ratio are associated with shorter progression-free survival in EGFR-mutant lung adenocarcinoma treated by EGFR tyrosine kinase inhibitor
Source: PLoS One. 2018 Sep 7;13(9):e0203625. doi: 10.1371/journal.pone.0203625 (PMC6128600; doi:10.1371/journal.pone.0203625)

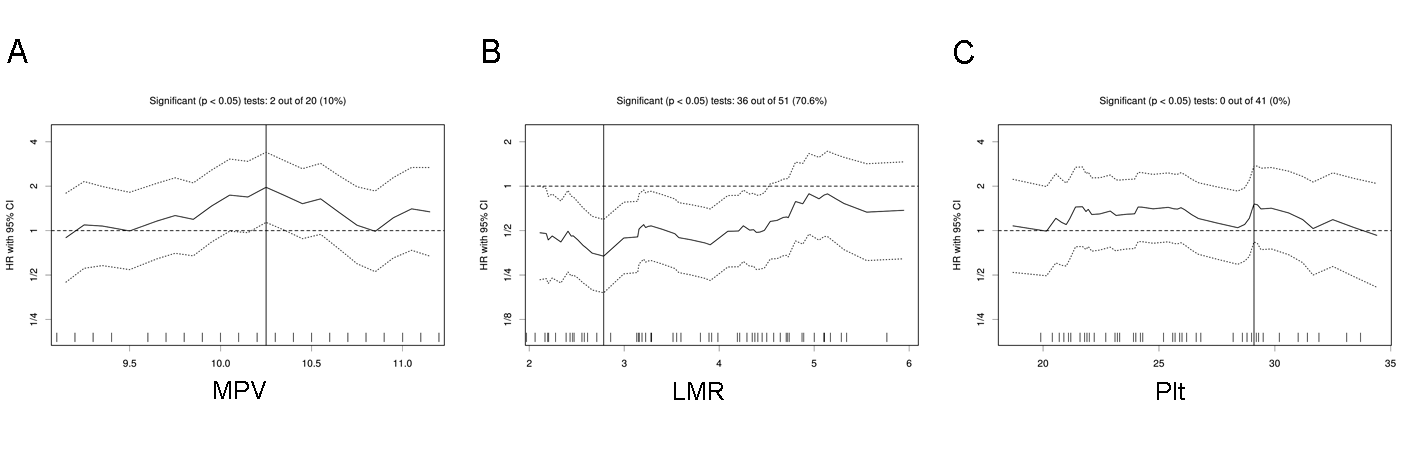

Supplement: S1 Fig — Hazard ratio (HR) for PFS with 95% CI is plotted in dependence of MPV (A), LMR (B) and platelet count (C). The distribution of each case is shown as rug plot at the bottom of the figure. A vertical line designates the cut-off point showing the most significant correlation with PFS. (TIF) [file pone.0203625.s001.tif]
